# RsmA Regulates *Aspergillus fumigatus* Gliotoxin Cluster Metabolites Including Cyclo(L-Phe-L-Ser), a Potential New Diagnostic Marker for Invasive Aspergillosis

**DOI:** 10.1371/journal.pone.0062591

**Published:** 2013-05-06

**Authors:** Relebohile Sekonyela, Jonathan M. Palmer, Jin-Woo Bok, Sachin Jain, Erwin Berthier, Ry Forseth, Frank Schroeder, Nancy P. Keller

**Affiliations:** 1 Department of Bacteriology, University of Wisconsin-Madison, Madison, Wisconsin, United States of America; 2 Department of Medical Microbiology and Immunology, University of Wisconsin-Madison, Madison, Wisconsin, United States of America; 3 Boyce Thompson Institute and Department of Chemistry and Chemical Biology, Cornell University, Ithaca, New York, United States of America; Geisel School of Medicine at Dartmouth, United States of America

## Abstract

Dimeric basic leucine zipper (bZIP) proteins are conserved transcriptional enhancers found in all eukaryotes. A recently reported and novel function for bZIPs is association of these proteins with secondary metabolite production in filamentous fungi. In particular a Yap-like bZIP termed RsmA (restorer of secondary metabolism A) was identified in *Aspergillus nidulans* that positively regulates the carcinogen sterigmatocystin. To assess for conserved function for RsmA, we examined a role of this protein in secondary metabolism in the pathogen *A. fumigatus.* RsmA was found to positively regulate gliotoxin where overexpression (OE) of *rsmA* led to 2–100 fold increases of twelve *gli* cluster metabolites in culture medium including the newly identified *gli* metabolite cyclo(L-Phe-L-Ser). Lungs from both wild type and *OErsmA* infected mice contained gliotoxin (2.3 fold higher in *OErsmA* treatment) as well as the gliotoxin precursor cyclo(L-Phe-L-Ser) (3.2 fold higher in *OErsmA* treatment). The data here presents a conserved role for RsmA in secondary metabolite cluster activation and suggests cyclo(L-Phe-L-Ser) may serve as an alternative marker for diagnosis of invasive aspergillosis.

## Introduction

*Aspergillus fumigatus* is an opportunistic pathogen of immunosuppressed individuals. In immunosuppressed hosts, invasive aspergillosis (IA) represents a major cause of morbidity and mortality, with mortality rates ranging from 40% to 90% [1], [2]. Part of the virulence of *A. fumigatus* is associated with its ability to produce a variety of toxic natural products. The natural product pathways in fungi result in the production of structurally diverse and low-molecular-weight compounds referred to as secondary metabolites (SMs) [3]. Although the functions of many fungal SMs remain to be determined, the known SMs of the genus *Aspergillus* have a tremendous impact on society. Compounds such as lovastatin (antihypercholesterolemic agent) and penicillin (antibiotic) are of clinical importance whereas the carcinogen, aflatoxin, causes both short term toxicities and long term liver cancer when humans eat contaminated crops [3].

Biosynthetic genes for individual SMs are typically clustered, and regulation of these clustered genes is often dependent on pathway-specific transcription factors. For example, the aflatoxin and related sterigmatocystin gene clusters contain one gene, *aflR*, that encodes a transcription factor regulating expression of the remaining biosynthetic genes [4], [5]; similarly *gliZ* encodes the transcription factor required for expression of the gliotoxin (*gli*) cluster genes in *A. fumigatus* [6]. In addition, SM cluster activation is also regulated at a higher hierarchic level by the nuclear protein LaeA [7]. Overexpression of *laeA* results in increase in the production of a multitude of SMs while deletion of *laeA* yields opposite results in many fungi [7]–[10]. Studies of the human pathogen, *A. fumigatus,* showed SMs such as gliotoxin are upregulated when *laeA* is overexpressed, and that deletion of *laeA* results in decreased virulence in both neutropenic and non-neutropenic murine models [6], [11].

The well-known *A. fumigatus* SM gliotoxin, a mycotoxin readily detected in IA, has immunosuppressive and apoptotic activities in the host [12]–[14]. Deletant strains of either *gliZ* or *gliP* (encoding a non-ribosomal peptide synthetase, NRPS, required for gliotoxin synthesis), are less damaging to host tissue during *in vitro* studies and insect model studies [15]–[17]. Additionally, these mutants show reduced virulence in non-neutropenic murine models but not neutropenic models [6], [18]–[20]. This toxin and/or its derivatives are also considered for use for diagnosis of IA [21], [22]. Aside from *gliZ,* no other transcription factors have been described to regulate the *gli* cluster.

Recently, several bZIP transcription factors have been found to regulate secondary metabolism in several fungal genera [23]–[27]. For example, a suppressor mutagenesis in *A. nidulans* identified *rsmA,* encoding a bZIP transcriptional enhancer, that when overexpressed could partially restore secondary metabolism in Δ*laeA* strains [28]. Overexpression of *rsmA* (*OErsmA*) increased sterigmatocystin production in *A. nidulans* more than 40 fold through transcriptional activation of *aflR* [29]. Here we find that overexpression of the *A. fumigatus rsmA* ortholog, also called *rsmA,* increases gliotoxin synthesis to a similar fold as sterigmatocystin in *OErsmA* strains of *A. nidulans.* This increase is mediated via transcriptional activation of *gliZ*. In addition to gliotoxin, the *gli* cluster derivative cyclo(L-Phe-L-Ser) was found to accumulate in murine lungs from both *OErsmA* and wild type infected mice and may represent an alternative marker for diagnosis of IA. Furthermore, supporting previous research data that indicates gliotoxin negatively impacts neutrophil migration [30] we found that supernatants of *A. fumigatus OErsmA* but not those of *OErsmA*Δ*gliZ* or wild type inhibit human neutrophil chemotaxis.

## Materials and Methods

### Ethics Statement

Neutrophils were obtained from whole blood of self-reportedly healthy donors, from which we obtained informed and written consent at the time of the blood draw with approval of the University of Wisconsin-Madison Center for Health Sciences Human Subjects committee. The Keller animal protocol number is M02468 as approved by the University of Wisconsin-Madison Research Animal Resources Center.

### Fungal Strains and Culture Conditions

The strains used in this study are listed in Table 1 and plasmids are listed in Table 2. All fungal strains were maintained as glycerol stocks, and cultured at 25°C or 37°C on glucose minimal medium (GMM) [31] with appropriate supplements.

**Table 1:** Strains of *Aspergillus fumigatus* used in this study.

Strains	Genotype	Source
AF293	Wild-type	[51]
AF293.1	*pyrG1*	[51]
AF293.6	*pyrG1, argB1*	[51]
TDWC5.6	*pyrG1,* Δ*gliZ::pyrG*	[43]
TJMP19.2	*pyrG1, argB1,* Δ*laeA::argB*	This study
TJMP36.2	*pyrG1, argB1,* Δ*laeA::argB, pyrG*	This study
TJMP54.16	*pyrG1, gpdA(p)::rsmA::pyrG*	This study
TJMP52.2	*pyrG1, argB1,* Δ*laeA::argB, gpdA(p)::rsmA::pyrG*	This study
TJMP56.6	*pyrG1, argB1, gpdA(p)::rsmA::pyrG*	This study
TRSR40.4	*pyrG1, argB1, gpdA(p)::rsmA::pyrG,* Δ*gliZ::argB*	This study
TRSR1.2	*pyrG1,* Δ*rsmA::pyrG*	This study

**Table 2:** Plasmids used in this study.

Plasmid	Genotype	Source
pTMH44.2	*gpdA(p)::GFP::trpC(t)*	[33]
pJW24	*A. parasiticus pyrG (3 kb)*	[34]
pJMP4	*A. fumigatus argB*	This study
pJMP7	*A. parasiticus pyrG (2 kb)*	This study
pJMP8	*A. nidulans gpdA promoter*	This study
pRS1	*gpdA(p)::rsmA::pyrG*	This study

### Nucleic Acid Analysis and Molecular Genetic Manipulations

Extraction of fungal DNA and RNA, restriction enzyme digestion, gel electrophoresis, Southern and northern blotting, hybridization and probe preparation were performed using standard methods [32]. Primers for PCR and probes are listed in Table 3.

**Table 3:** Oligonucleotides used in this study.

Primer Name	Sequence (5′-3′)	Purpose
JP Afumi argB For	GAACGCGGTCTGCATCCAAG	Create pJMP4/argB
JP Afumi argB Rev	GAAGGAGAGACCCATACATCC	Create pJMP4/argB
JP Apara pyrG Short For	GCTTGATATCGAATTCTCATGTTTG	Create pJMP7
JP Apara pyrG Short Rev	TACGGAAGCTTATACGAACAGATGG	Create pJMP7
JP gpdA(p) EcoRI For	TTTCGAATTCCATCCGGATGTCGAAGG	Create pJMP8 and pRS1
JP gpdA(p) BamHI Rev	CTTGTGGATCCCGTCATCTTTGAAA	Create pJMP8
MK Afumi Bzip Fusion Rev	ATTGGAATAAAATGAGTAGTCCATGGTGATGTCTGCTCAAGCGG	Create pRS1
MK Afumi Bzip Fusion For	CCGCTTGAGCAGACATCACCATGGACTACTCATTTTATTCCAAT	Create pRS1
MK Afumi Bzip rev (EcoRI)	GTTGTACTAGAAGAGAATTCGCAGTC	Create pRS1
RS rsmA 5′F For	GTGAAGACGGCAATCTCTGAG	ΔrsmA 5′ flank
RS rsmA 5′ F+pyrg tail Rev	CCCTATAGTGAGTCGTATTACGATCTGTGCACTTCCCTCAAC	ΔrsmA 5′ flank
RS pyrG +3′F rsmA For	CGATGATAAGCTGTCAAACATGAGCCTTGGCAGGGCGAATTTGG	ΔrsmA 3′ Flank
RS rsmA 3′ F Rev	CAGCAAATCGTTCGTGCTCG	ΔrsmA 3′ Flank
RS rsmA screen For	GATCTACAACGCTTCGACGGAC	PCR screen rsmA
JP Af-laeA 5′ Fl For	CCTCGTTGAACTAATTGGCG	ΔlaeA 5′ Flank
JP Af-laeA 5′ Fl Rev	GGAAAATTTGTCTTGGATGCAGTCCGCGTTCAGGTCGAGGAGGTCCAATC	ΔlaeA 5′ Flank
JP Af-laeA 3′ Fl For	AGATCAAATGGATGTATGGGTCTCTCCTTCGCTTCAAACCTCTGAGATCG	ΔlaeA 3′ Flank
JP Af-laeA 3′ Fl Rev	CGACACACATATCATGACGG	ΔlaeA 3′ Flank
FY GZ 5′F FOR	GAGTTCTCGCTAGTGTCTGG	ΔgliZ 5′ Flank
RS ArgB tail +5′Rev	GTCTTGGATGCAGACCGCGTTCCGCAGTGCAACACGGGTGGC	ΔgliZ 5′ Flank
RS GliZ 3′ Fl For	CGCAGTGCAACACGGGTGGCGTCTTGGATGCAGACCGCGTTC	ΔgliZ 3′ Flank
FY GZ 3′F REV	CGAGTCAACATGATGACGG	ΔgliZ 3′ Flank
RS gliZ 5′ For	TGAGCCATACTGAAGTCTCCGTG	ΔgliZ nested
RS gliZ 3′ Rev	GACATCGACTGAACATGTCCACG	ΔgliZ nested
RS Af RsmA Int For	CAATCCTCAGTCACAGCAGC	rsmA probe
RS Af RsmA Int Rev	GGTCATATCAATTCATCGCGGC	rsmA probe
JP Modified T7 Promoter For	CGTAATACGACTCACTATAGGG	Amplify pyrG from pJMP7
RS A.para pyrG Rev	CTCATGTTTGACAGCTTATCATCG	Amplify pyrG from pJMP7
RS rsmA RT-PCR For	ACCGAGCTGCTCAGCGAGCG	rsmA RT-PCR
RS rsmA RT-PCR Rev	CTGGCCCTCCCTGAACGCCG	rsmA RT-PCR
RS actin RT-PCR For	GTCACCATGGTATCATGATTGG	actin RT-PCR
RS actin RT-PCR Rev	GGTAGTCCGTCAGATCACGG	actin RT-PCR
RS laeA RT-PCR For	CCAGTAGCAAGAATCCTGAC	laeA RT-PCR
RS laeA RT-PCR Rev	CCGGAGCCCATCTGCATGTG	laeA RT-PCR
RS gliZ int For	GCACCTACAGCTATTCCTCG	gliZ probe
RS gliZ int Rev	GTCACGGCCATGCTAATACTG	gliZ probe
RS actin int For	GTATGTCGGTGATGAGGCAC	Actin probe
RS actin int Rev	CCGTAGAGATCCTTACGGAC	Actin probe
GIINTF	TGTTGATCGAGACGCCGTTCTG	gliI probe
GIINTR	CAGAGCGGCTCGATTCTGGTG	gliI probe
RS gliT For	GCAAACTACTCTCCAACGGAG	gliT probe
RS gliT Rev	GCAAACTACTCTCCAACGGAG	gliT probe

### Construction of Plasmids

A 1.5-kb PCR fragment of the *A. nidulans gpdA* promoter was amplified from pTMH44.2 [33] using primers JP gpdA(p) EcoRI For and JP gpdA(p) BamHI Rev and subsequently cloned into pBluescript II SK- (Fermentas) to generate pJMP8. A plasmid containing the selectable marker gene for complementation of the *argB1* allele in *A. fumigatus* was generated by PCR amplifying *argB* (Afu4g07190) from gDNA of AF293 with the primers JP Afumi argB For and JP Afumi argB Rev, the resulting PCR product was cloned into pCR-Blunt II-TOPO (Invitrogen) to create pJMP4. To construct a *pyrG1* complementation plasmid, a 2.0-kb *A. parasiticus pyrG* PCR product was amplified from pJW24 [34] using primers JP Apara pyrG Short For and JP Apara pyrG Short Rev and subsequently cloned into pBluescript II SK- (Fermentas) to generate pJMP7. A plasmid to over-express *rsmA* was created by fusion PCR [35] of the gpdA promoter (JP gpdA(p) EcoRI For and MK Afumi Bzip Fusion Rev) and a 1.5-kb *rsmA* product (MK Afumi Bzip Fusion For and MK Afumi Bzip EcoRI Rev). The resulting 3.0-kb fusion PCR product was cloned into pJMP7 with *EcoRI* to generate pRS1.

### Construction of Fungal Strains

Fungal transformation was done as previously described [36]. Gene replacement constructs for *laeA*, *rsmA*, and *gliZ* were made using double-joint PCR [35], [37]. Briefly, approximately 1-kb flanking regions were PCR amplified from gDNA of AF293 and fused to either the *A. fumigatus argB* gene from pJMP4 or the *A. parasiticus pyrG* gene from pJMP7 (primers are listed in Table 3). The *laeA* gene was disrupted with *A. fumigatus argB* in the AF293.6 (*pyrG1, argB1*) background to create TJMP19.2 (Δ*laeA, pyrG1*) and TJMP19.2 was subsequently transformed with pJMP7 to create the prototrophic TJMP36.2 (Δ*laeA*) strain. These strains were confirmed by Southern blot analysis (data not shown). Deletion of *rsmA* was achieved by replacement with the *A. parasiticus pyrG* gene in AF293.1 (*pyrG1*) to generate TRSR1.2 which was confirmed by Southern blot (Figure S1B). Over expression of *rsmA* (*OErsmA*) was achieved by transformation of pRS1 into AF293.1, TJMP19.2, and AF293.6, which created TJMP54.16 (*OErsmA*), TJMP52.2 (Δ*laeA*, *OErsmA*), and TJMP56.6 (*OErsmA*, *argB1*) respectively (Figure S1A). To create a double *OErsmA*Δ*gliZ* strain, *gliZ* was replaced with the *A. fumigatus argB* gene in TJMP56.6 resulting in TRSR40.4 (Figure S1C).

### Gene Expression Analysis

10^7^ fungal spores/ml were grown in liquid GMM at either 37°C, 250 rpm for 48 h or 25°C, 280 RPM for 72 h and total RNA was extracted via Isol-RNA Lysis Reagent (5 Prime) as previously described [36]. For semi-quantitative reverse transcriptase PCR, cDNA was generated using the iScript kit (Bio Rad) following manufacturers recommendations.

### Physiological Studies

10^4^ conidia from strains were point-inoculated onto solid glucose minimal media (GMM) in four replicates and incubated in continuous dark at 25°C for 12 days and at 37°C for 4 days. Radial growth was assayed by measuring the diameter of point-inoculated colonies after 4 days and 12 days incubation at 37°C and 25°C, respectively. Conidia were counted from 2 cm agar cores taken from the center of the point-inoculated cultures and enumerated using the hemocytometer.

### Secondary Metabolite Analysis

Secondary metabolite production was assessed by thin-layer chromatography (TLC) from cultures grown in liquid glucose minimal medium (GMM) for 3 days at 25°C and at 280 rpm in triplicate. Secondary metabolites from the organic layer were extracted with chloroform and the air-dried extracts were resuspended in 100 µl of methanol and resolved in chloroform: acetone (7∶3) on UV infused TLC plates. Gliotoxin (Sigma) was spotted as standard. The TLC plates were then visualized at 366 nm and 254 nm.

### HPLC-MS Profiling of Compounds 1–13 in Wild Type and *OErsmA* Cultures

(**a**) Liquid culture preparation: 10^7^ fungal spores/ml were grown in triplicates in liquid GMM (50 mL) at 25°C, 280 rpm for 72 h. Three 50 mL liquid culture supernatant samples of both wild type and *OErsmA* were frozen and lyophilized. To each of the lyophilized samples, 50 mL of a mixture consisting of 80% acetonitrile, 15% ethyl acetate, and 5% H_2_O was added. After stirring for 2 h, the mixtures were filtered over extraction solvent-washed cotton. The extraction process was repeated twice more using 10 mL of the extraction solvent. The solvent was removed using rotary evaporation. (**b**) Liquid culture analysis: The evaporated samples were dissolved in 250 µL of 10% water in acetonitrile, and 2 µL of the resulting solution was injected into an Agilent 1100 series HPLC. The HPLC system, equipped with an Agilent Zorbax Eclipse XDB-C8 column (4.6×150 mm, 5 µm particle diameter), running a water-acetonitrile gradient (2 mL/min) starting with 5% acetonitrile for 5 min, followed by a linear increase to 36% acetonitrile over 25 min, followed by a rapid linear increase to 100% over 10 min, and ending with 10 min at 100% acetonitrile. The eluent of the HPLC was split (1∶10) into a Quattro II electrospray ionization (ESI) mass spectrometer operated in ESI positive (ESI^+^) ionization mode. Single ion monitoring (SIM) was used to increase sensitivity by monitoring the major ions produced by each analyte. Enriched samples of **1–13** served as standards to determine retention time and ionization/fragmentation for each analyte (enriched standards were obtained as described [38]). (**c**) Data processing: Ion chromatogram peak areas were measured for each analyte and used to assess the relative amounts in *OErsmA* compared to wild type.

### Oxidative Stress Tolerance Assays

For studying the relative sensitivities of wild type and mutant strains to oxidative stress, 10^5^ conidia in 5 µl were inoculated on GMM with 20, 30 and 40 µM menadione. Menadione was filter sterilized and added to agar medium at ∼50°C before solidification. Plates were incubated at 37°C for 48 h. GMM without menadione served as a control.

### Neutrophil Recruitment

Neutrophil recruitment assays were performed using a microfluidic chemotaxis platform developed and described in more detail elsewhere [39]. In brief, the device is able to generate a linear gradient of soluble factors between a source channel containing the compounds to be tested and a sink channel containing the neutrophils. The gradient is generated in a thin cross-channel in which neutrophils from the sink channel can invade. The quantification was performed using software developed in-house (source code and software can be obtained upon request). The neutrophil purification was performed with a gradient centrifugation process using Polymorphprep solution, according to the manufacturer’s recommendations (Nycomed, Sheldon, UK). The neutrophils were re-suspended to a concentration of 4 million per mL in PBS, and kept no more than 2 h at 4 degrees Celsius. The microfluidic devices were prepared by filling them with modified HBSS (HBSS solution containing 2% HEPES buffer and 0.1% HAS), after which 4 µL of neutrophil suspension was added in each sink channel of each device, and finally 3 µL of the crude supernatants was inserted into each source channel of each device. The negative control was performed by adding blank GMM (the fungal growth medium) to the source channel, and the positive control was performed by adding a solution of 100 nM fMLP (F3506, Sigma Aldrich) in GMM.

### Animal Model of *Aspergillus* Infection

**(a)** *A. fumigatus* wild type and *OErsmA* strains were assessed in a lung infection model of invasive aspergillosis. Briefly, female Swiss ICR mice (Harlan Sprague Dawley) weighing about 18–20 g were immunosuppressed via administration of cyclophosphamide by separate intraperitoneal injections, one at 4 days (200 mg/kg of body weight) and the other at 1 day (200 mg/kg) before infection. The treatment included administration of cortisone acetate at a dose of 250 mg/kg by separate intraperitoneal injection at 1 day before infection. Anesthetized mice (10 mice/fungal strain) were infected by nasal instillation of 50 µl of 1×10^7^ conidia/ml (day 1) and monitored three times daily for 7 days postinfection. The control group was inoculated with saline (0.85% NaCl). All surviving mice were sacrificed at day 7. Health and comfort of animals was assessed twice a day by monitoring feeding and water intake. Animals were housed in the Microbial Science Building Vivarium. If animals were ill (decreased activity, difficulty moving, ruffled fur, decreased movement with stimulation) they were euthanized via CO_2_ inhalation. **(b)** Assessment of presence or absence of compounds **1–13** in the lungs of mice infected with wild type and *OErsmA*: Seven mice were infected per *A. fumigatus* strain as described above. Dissected lungs were lyophilized, and powdered using a metal spatula. Lung samples were extracted with 50 mL of acetonitrile and filtered over acetonitrile-washed cotton. Solvent was removed using rotary evaporation. Samples were redissolved in 20 µl acetonitrile, and 2 µL was injected into the HPLC-ESI^+^-SIMMS system described above. The chromatographic gradient consisted of water-acetonitrile starting with 5 min 5% acetonitrile, followed by a linear increase to 20% acetonitrile over 15 min, followed by a linear increase to 100% acetonitrile over 10 min, ending with 10 min at 100% acetonitrile. Enriched samples of **1–13** served as retention time and ionization standards.

### Statistical Analysis

Statistical differences were analyzed using the JMP software package version 3.2.6 (SAS Institute, Inc., Cary, NC). Multiple comparisons of results for all strains were calculated for growth diameter and sporulation. Statistically significant mean values, indicated with different letters in the figures, are significant at *P*<0.0001.

## Results

### *rsmA* is a Low Expressed Gene

After confirming the creation of both the deletion of *rsmA* and *gliZ* and overexpression of *rsmA* in the appropriate backgrounds (Figure S1), northern blot analysis was carried out to assess *rsmA* expression in all strains. mRNA transcript level was barely detectable in wild type (WT, Figure S2A) and semi-quantitative RT-PCR was employed for confirmation of expression (Figure S2B). The *OErsmA* mutant showed multiple fold increase in mRNA transcript level, verifying the overexpression construct (Figure S2). The low expression of *rsmA* in wild type has also been characterized in *A. nidulans* [28].

### *OErsmA* Exhibits a Growth Defect at Low Growth Temperature

**Figure 1:**
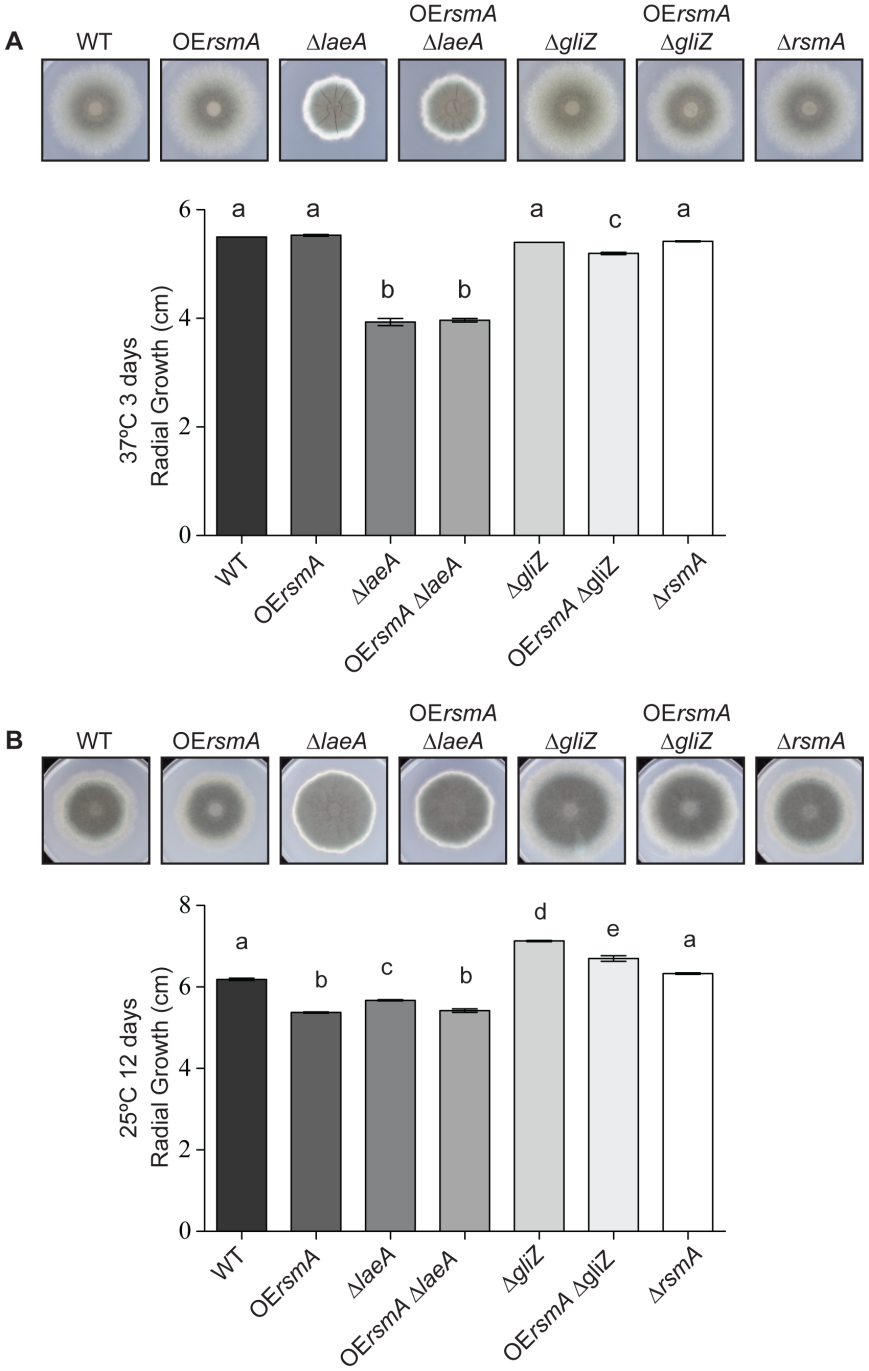
Average radial growth of *A. fumigatus* strains. 10^4^ conidia of each strain were point inoculated on GMM and grown at 37°C for 4 days and at 25°C for 12 days (Figure 1). Radial growth was measured at the end of each growth period. Means ± standard deviations are indicated for four replicates of each strain. Levels not connected by same letter are significantly different (*P*<0.0001) according to ANOVA analysis.

At 37°C, the radial growth of *OErsmA* strain is similar to that of wild type (Figure 1A). However, at 25°C, the radial growth of *OErsmA* is decreased compared to that of the wild-type strain (Figure 1B). Similar results were observed for the *OErsmA*Δ*gliZ* and *OErsmA*Δ*laeA* strains, with a significant decrease in radial growth at 25°C compared to the Δ*gliZ* and Δ*laeA* control strains. The radial growths of strains in the Δ*laeA* background were decreased at both temperatures compared to wild type. No significant differences were observed in spore production between Δ*rsmA*, the *OErsmA* background strains and their respective controls (Figure S3).

### *OErsmA* Mutants are Resistant to Menadione

**Figure 2:**
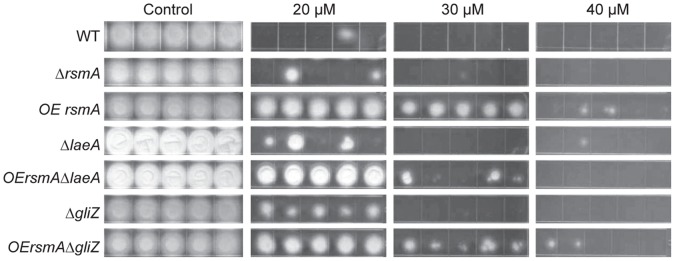
*OErsmA* mutants are resistant to menadione. For each strain, 10^5^ conidia in 5 µl were spotted on GMM plates with 20 µM, 30 µM and 40 µM of menadione, or on GMM only for a control. Each strain was replicated 5 times. The plates were incubated at 37°C for 48 h.

An examination of RsmA function in *A. nidulans* showed that RsmA binds a Yap1 DNA-binding site [29]. Although *A. nidulans* RsmA showed no response to oxidative stress [40], other fungal Yap-like bZIP proteins are often associated with the reactive oxygen species (ROS) response [41], [42]. To assess any possible role of *A. fumigatus rsmA* on oxidation stress, conidia of *OErsmA, OErsmA*Δ*laeA*, *OErsmA*Δ*gliZ* and Δ*rsmA* mutants and their controls were inoculated on GMM with varying concentrations of menadione and incubated at 37°C for 48 h. All strains containing an *OErsmA* allele grew better than their respective controls on 20 and 30 µM menadione, regardless of the absence of either the *laeA* or *gliZ* allele, thus showing increased resistance of these strains to ROS induced by menadione (Figure 2).

### *OErsmA* Increases Production of Multiple *gli* Pathway Metabolites

In *A. nidulans,* overexpression of *rsmA* increased the production of sterigmatocystin 40–100 fold dependent on culture condition [28]. To examine any possible conserved role of RsmA in *A. fumigatus*, we examined SM production in several cultural conditions. First strains were grown in gliotoxin inducing conditions (liquid GMM for 3 days, shaking at 25°C). Here the *OErsmA* strain produced more gliotoxin than wild type, and no gliotoxin was visibly detected in Δ*laeA* background strains by thin layer chromatography, nor was gliotoxin production rescued in the *OErsmA*Δ*laeA* double mutant (Figure S4). As expected the increase of gliotoxin was dependent on presence of the *gliZ* gene as observed by gliotoxin loss in the *OErsmA*Δ*gliZ* mutant. By eye, Δ*rsmA* produced a similar TLC profile as wild type. Extracts from strains grown at 37°C on solid medium did not exhibit noticeable differences in SM profiles (data not shown).

**Figure 3:**
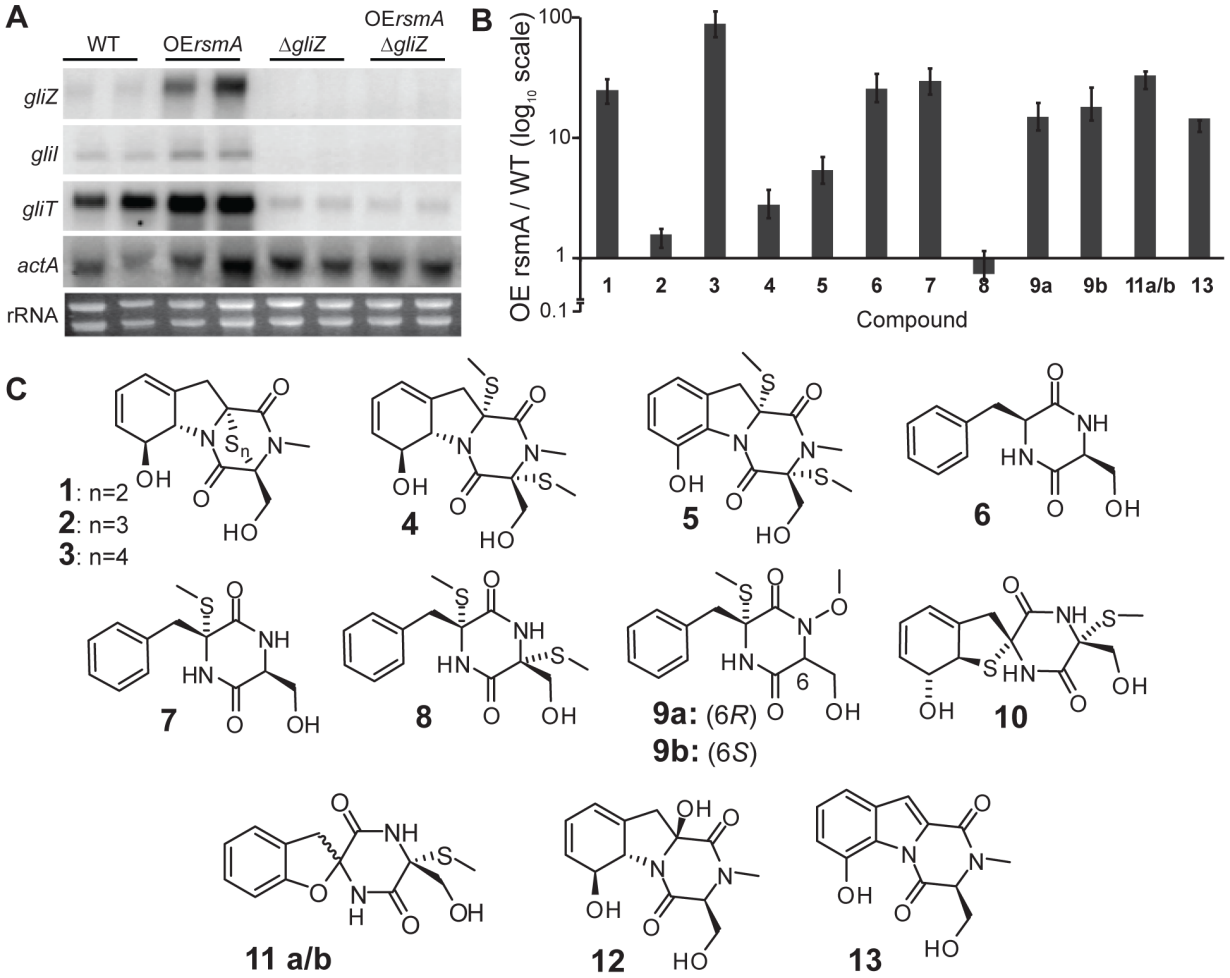
RsmA regulates production of gliotoxin and is dependent on *gliZ*. **A.** *gliZ, gliI* and *gliT* gene expression in liquid shaking cultures. *gliZ, gliI* and *gliT* expression in *A. fumigatus* (AF293) wild type, *OErsmA*, *ΔgliZ*, and *OErsmAΔgliZ.* All strains were grown in 50 mL GMM liquid shaking culture at 25°C and 280 rpm for 72 h. Total RNA was extracted from mycelia and probed for *gliZ, gliI* and *gliT.* WT = wild type. **B.** Relative amounts of compounds **1**–**13** in *OErsmA* compared to WT. Compounds **10** and **12** were not detected in the *OErsmA* nor WT cultures. Compounds **11a/b** and **13** were not detected in wild type, their values represent the measured S/N ratio as determined by their diagnostic mass ion chromatograms. Data was triplicated and the error bars represent 1 standard deviation. **C**. Collection of *gliZ*-dependent metabolites profiled in this study, structures and *gliZ*-dependency in *A. fumigatus* was previously established in [38].

An examination of *gliZ, gliI* (a biosynthetic gene in the *gli* cluster) and *gliT* (encoding a gliotoxin oxidase providing endogenous protection against gliotoxin) expression at gliotoxin inducing conditions verified a positive impact of *rsmA* on gliotoxin gene expression where all three genes were upregulated in the *OErsmA* background (Figure 3A).

**Figure 4:**
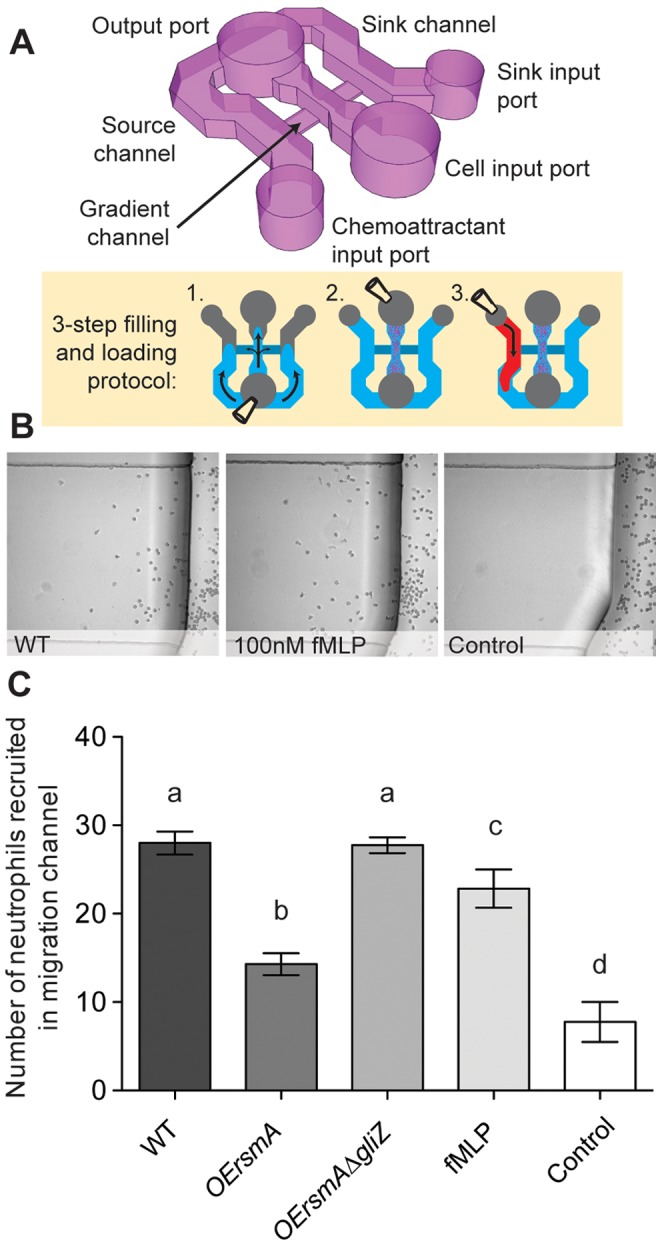
Neutrophil chemotaxis is reduced when exposed to extracts from *OErsmA*. **A.** Schematic of the neutrophil migration platform used. The neutrophils are placed in the center channel; the compound to be tested is placed in one of the side channels, while the other channel acts as a negative control. The device is prepared in 3 steps: (1) filling, (2) adding neutrophils, and (3) adding the fungal culture supernatants. **B**. Representative microscopy images of the migration area after 1 h incubation. Supernatant of wild type *A. fumigatus* (AF WT) has chemoattractive properties on par with the known chemoattractant fMLP. **C**. Quantification of neutrophil recruitment to the crude supernatant of wild type, *OErsmA*, and *OErsmAΔgliZ* strains (significant differences at P<0.5 are indicated by different letters).

The increased expression of *gliZ* in the *OErsmA* strain was of considerable interest as GliZ has been found to be required for the production of a multitude of *gli* cluster metabolites, including the family of diketopiperazines shown in Figure 4C (**1**–**13)** [38]. We found that compounds **6**, **7**, **9**, **11**, **13,** and the gliotoxins **1** and **3** are at least 10-fold up-regulated in *OErsmA*, whereas the methylsulfanyl containing compounds **4**, **5**, and **8** are much less or not at all upregulated (Figure 4B). The methylsulfanyl groups in compounds **4**, **5**, and **8** likely arise from SAM-dependent methylation of free thiols. Their relatively lower abundance compared to the other compounds in *OErsmA* logically follows that of overexpression of *gliT* in *OErsmA* (Figure 3A), the gene encoding the oxidase responsible for disulfide formation from dithiol-containing metabolites. Overexpression of *gliT* would be expected to prevent the accumulation of large amounts of free thiols. We further found that production of the four sulfur atom-containing gliotoxin G (**3**) is dramatically increased in *OErsmA* whereas the three sulfur atom-containing gliotoxin E (**2**) is only modestly up-regulated, suggesting that the biosynthesis of different gliotoxin derivatives may be regulated independently.

### *gli* Cluster Metabolites Decrease Neutrophil Migration

As an earlier study had suggested gliotoxin inhibited neutrophil chemotaxis [30], the supernatants from wild type, *OErsmA* and *OErsmA*Δ*gliZ* were assessed for the ability to alter neutrophil migration. Results show that neutrophils are strongly recruited to the supernatant of *A. fumigatus* wild type, in a magnitude on par with the recruitment towards a known chemotactic compound, fMLP at 100 nM (Figure 4). The supernatant of *OErsmA*, however, induces a significant decrease in neutrophil migration. The decrease is rescued in the *OErsmA*Δ*gliZ* strain. These results suggest that gliotoxin, or a metabolite in the gliotoxin pathway, is the cause of the neutrophil migration defect observed in the over-expressing *rsmA* strain.

### Detection of Cyclo(L-Phe-L-Ser) in Murine Infection

**Figure 5:**
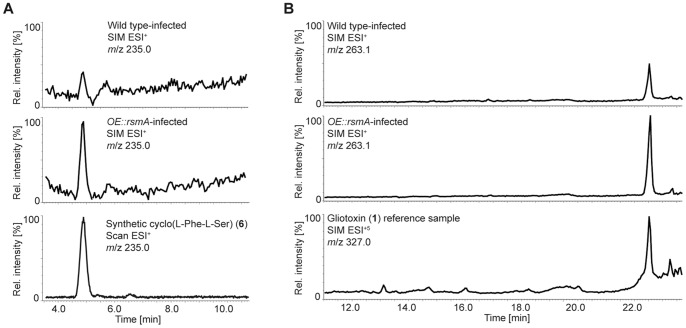
Infected mouse lung extracts (IMLE) HPLC- single ion monitoring (SIM)MS ion chromatograms. HPLC-SIMMS analysis of crude mouse lung extracts corresponding to mice infected with wild type, or *OErsmA*. (**A**) Ion chromatogram showing compound **6** is approximately two to three times as abundant in the *OErsmA*-IMLE relative to WT-IMLE. (**B**) Similarly, gliotoxin is about two times as abundant in *OErsmA*-IMLE than WT-IMLE. Reference chromatograms (bottom panels) show diagnostic ions of cyclo(L-Phe-L-Ser) (**6**) and gliotoxin (**1**). Lung extract chromatograms are scaled to the peaks measured in the *OErsmA*-IMLE sample (bottom panels of standards are not to scale).

Gliotoxin has previously been isolated from the murine lung environment [43], however it is unknown if gliotoxin intermediates also accumulate *in vivo*. To test this hypothesis, we conducted a neutropenic IA mouse assay with wild type and *OErsmA* strains and assayed for the presence or absence of compounds **1–13** from murine lungs. There was no significant difference in virulence between wild type versus *OErsmA* in the neutropenic model of IA (Figure S5). Lungs from wild type and *OErsmA*-infected mice were homogenized and extracted and HPLC-ESI^+^-SIMMS analysis showed that gliotoxin (**1**) and cyclo(L-Phe-L-Ser) (**6**), were present in lung tissue from both wild type and *OErsmA* treated mice. Both compounds were present in higher amounts (2- and 3-fold, respectively) in *OErsmA* lungs (Figure 5A and 5B). Compound **6** has not previously been observed in *A. fumigatus*-lung infection models. The absence of the remaining *gliZ*-dependent compounds, **2**–**5** and **7**–**13** in the wild type and *OErsmA*-infected lung tissue suggests that these compounds are not produced in amounts large enough for detection or that they were not detected due to decomposition, as the methylsulfanyl groups in **4**, **5**, and **7**–**11** are easily substituted by other nucleophiles, including water [38].

## Discussion

Our interest in exploring RsmA in impacting secondary metabolism in *A. fumigatus* arose from studies using the model genetic system, *A. nidulans*. RsmA (restorer of secondary metabolism A) was identified in a mutagenesis screen of an *A. nidulans ΔlaeA* strain where overexpression of *rsmA* (*OErsmA*) partially rescued secondary metabolism in a *ΔlaeA* genetic background and greatly over produced the carcinogen sterigmatocystin in a *laeA* wild type background [28]. LaeA, a conserved protein in filamentous fungi, governs the production of multiple SMs that contribute to virulence in all pathogenic fungi examined to date including *A. fumigatus* [6], [8], [10], [44]. Here we found that although *OErsmA* did not restore gliotoxin synthesis in the *ΔlaeA* background in *A. fumigatus, rsmA* overexpression did have a profound effect on *gli* cluster gene expression and concomitant metabolite production, mediated by GliZ, the *gli* gene cluster transcription factor.

In *A. nidulans*, RsmA binds to the promoter and activates expression of *aflR*, encoding the transcription factor required for sterigmatocystin and aflatoxin synthesis [29]. The *A. fumigatus* RsmA ortholog shares overall 58% identity to that of *A. nidulans* and 100% identity in the DNA binding region suggesting that both proteins bind to the same DNA sites, several of which are found in the *gli* gene cluster and possibly involved in the activation observed in this study. Several bZIP proteins have recently been found to regulate fungal SM clusters – both positively and negatively - including the bZIPs AtfB and ApYap1 regulating aflatoxin in *A. parasiticus* [24], [25], AoYap1 regulating ochratoxin in *A*. *ochraceus* [23], MeaB regulating bikaverin in *Fusarium fujikuroi* [26] and BcAft1 regulating botrydial, botryendial and botcinin A in *Botrytis cinerea* [27]. A distinguishing characteristic of RsmA, in both *A. nidulans* and *A. fumigatus*, is the extreme up-regulation of a primary SM in each species. In *A. nidulans OErsmA* led to 40-fold induction of sterigmatocystin, an anti-predation metabolite protecting this species from fungivory [29]. Here *OErsmA* increased production of a multitude of GliZ dependent metabolites 10 to near 100 fold over wild type. Gliotoxin is a potent anti-fungal thought to help establish a niche for *A. fumigatus* in natural settings [45]; it may be that RsmA represents a bZIP pathway hardwired for defensive SM production in these species [29].

One difference between *OErsmA* in *A. nidulans* and *A. fumigatus* was the role of this protein in the stress response of the two species. bZIP factors are associated with the stress response in fungi, such as NapA in *A. nidulans* [41] and *Afyap1* in *A. fumigatus* [42], [46]. Despite the lack of a stress response in the *A. nidulans rsmA* mutants [40], the *A. fumigatus* strains containing an *OErsmA* allele showed increased resistance to menadione, an indicator of resistance to reactive oxidative species (ROS), than the control strains. This was irrespective of presence of *gliZ* or *laeA* (Figure 2), the latter result in contrast to the non-affect of *OErsmA* on secondary metabolism in the *ΔlaeA* background. These observations suggest that RsmA directly or indirectly activates genes that are involved in oxidative stress response of *A. fumigatus.* In the animal host, the fungus faces a variety of toxic environmental challenges, which are aimed at eliminating the fungus. For example, neutrophils produce high levels of reactive oxygen species (ROS) during oxidative burst. However, pathogenic fungi possess oxidative-stress response genes that help to evade the otherwise lethal elevation of ROS. However, because the *OErsmA* strain showed no difference compared to the WT in the murine model, this factor of enhanced resistance to ROS likely has small impact on virulence of *OErsmA.* This is reminiscent of the *A. fumigatus Afyap1* mutant which while Afyap1 is important for resistance to ROS, the deletant strain did not exhibit a pathogenicity defect [42]. Possibly these two proteins play a redundant role in *A. fumigatus* ROS resistance.

The inhibition of neutrophil chemotaxis with *OErsmA* extracts supports earlier studies where gliotoxin or *A. fumigatus* extracts were found to diminish PMN chemotaxis [30], [47]. Currently the mechanism of this impact of chemotaxis is not known and could simply be attributable to the apoptotic properties of gliotoxin and, perhaps, other *gli* cluster metabolites. The great increase in *gli* cluster metabolites as observed in culture grown *OErsmA* was tempered in the animal model (ca. 10 fold less increase in murine lung). This may explain differences in the results from the *in vivo* (no difference in pathogenicity in the murine model) and *in vitro* (inhibition of chemotaxis) results of the *OErsmA* strain in this study.

In terms of *gli* cluster regulation, the greatly enhanced production of numerous *gli* cluster metabolites in *OErsmA* was striking and supported and extended our earlier studies showing that gliotoxin is not the only metabolite produced by the *gli* gene cluster [38]. Here we show that in addition to gliotoxin, cyclo(L-Phe-L-Ser) (**6**) accumulates in lungs of IA mice. Two pertinent observations from this study include further support for GliT as an oxidase responsible for disulfide formation from dithiol-containing metabolites. Notably, the lesser accumulation of the methylsulfanyl containing compounds (**4**, **5**, and **8)** would be expected in the *OErsmA* as *gliT* expression was particularly upregulated in this strain. GliT is also responsible for *A. fumigatus* self-resistance to gliotoxin, and likely other *gli* metabolites [48], [49] and, along with bis(dethio)bis(methylthio)gliotoxin (**4)** [22], has been proposed as an antigen for diagnosis of IA [50]. Our work here reveals an additional metabolite, cyclo(L-Phe-L-Ser) (**6**), that could serve as a marker for IA diagnosis. This compound, along with gliotoxin, is produced in high amounts by the fungus both *in vivo* and *in vitro* and, at least *in vitro*, is chemically more stable than gliotoxin or bis(dethio)bis(methylthio)gliotoxin (**4**).

## Supporting Information

Figure S1 **Overexpression and deletion of** ***rsmA*** **, and deletion of a transcription factor of gliotoxin biosynthesis, gliZ. A.** Southern blot analysis of the *OErsmA*Δ*laeA, OErsmA* and wild type (WT) strains. Genomic DNA was digested with KpnI. Expected hybridization band patterns: 3.5 kb and 5.6 kb for mutants, and 3.5 kb for wild type strain. **B.** Southern blot analysis of the Δ*rsmA* mutant strains and wild type (WT) strain. Genomic DNA was digested with BglI and PvuII. Expected hybridization band patterns: 5.572 kb (BglI), 4.571 kb and 2.457 kb (PvuII) for mutants; 1.633 kb and 3.149 kb (BglI), 6.038 kb (PvuII) for wild type strain. **C.** Southern blot analysis of the Δ*gliZ* and *OErsmA*Δ*gliZ* mutant strains and wild type(WT) strain. Genomic DNA was digested with SpeI and EagI. Expected hybridization band patterns: 5.17 kb (SpeI) and 6.21 kb (EagI) for mutants; 4.03 kb (SpeI), 1.13 kb and 3.96 kb (EagI) for wild type strain. The arrowheads denote the correct mutants.
(TIF)

Figure S2 **Detection of** ***rsmA*** **by Northern blot and reverse transcriptase- PCR (RT-PCR).** **A.** Northern blot analysis of *A. fumigatus* wild type (WT), Δ*laeA*, *OErsmA*Δ*laeA* and *OErsmA* strains. 10^7^ spores/ml were inoculated in triplicates into liquid GMM and incubated at 37°C shaking for 48 h. Mycelia were collected, frozen in liquid nitrogen and lyophilized overnight. Total RNA was isolated. An internal fragment of the *rsmA* open reading frame was used as a probe. As a control, RNA blots were also hybridized with an internal fragment of the *gpdA* gene. **B**. Reverse transcriptase-PCR of wild type (WT), Δ*laeA*, *OErsmA*Δ*laeA* and *OErsmA* strains. RNA of each strain was extracted from freeze-dried mycelia, treated with DNase1, reverse transcribed and the resultant cDNA was amplified with primers specific for *rsmA*, *laeA* and *actin* (serves as a control).
(TIF)

Figure S3 **Average sporulation of** ***A. fumigatus*** **strains.** 10^4^ conidia of each strain were point inoculated on GMM and grown under dark conditions at 25°C for 12 days (**A**) and at 37°C for 4 days (**B**). Agar plugs were extracted from the center of the colonies, homogenized in water and spores counted using a haemocytometer. Means ± standard errors are indicated for four replicates of each strain. Levels not connected by same letter are significantly different (P>0.15) according to the student’s t test.
(TIF)

Figure S4 **Thin-layer chromatography profiles of secondary metabolites produced by** ***A. fumigatus*** **wild type (WT),** ***OE rsmA*** **,** Δ***laeA*** **,** ***OE rsmA***Δ***laeA,*** Δ***gliZ, OE rsmA***Δ***gliZ*** **and** Δ***rsmA*** **strains.** Secondary metabolites were extracted by chloroform from cultures grown in liquid GMM at 25°C, 280 rpm for 3 days. Dried extracts were resuspended in 100 µl of methanol, and 10 µl was used for separation on TLC plate. All strains were triplicated. The solvent condition was chloroform:acetone (7∶3), and the plates were visualized at 254 nm (top) and 366 nm (bottom). G, gliotoxin standard.
(TIF)

Figure S5 **Virulence of** ***OErsmA*** **in a murine lung infection model.** 10 female Swiss ICR mice immunosuppressed by intraperitoneal injection of cyclophosphamide (200 mg/kg) and cortisone acetate (250 mg/kg) were inoculated intranasally with 50 µl of 1×10^7^ conidia/ml of *A. fumigatus* wild type (AF293) and *OErsmA* and control (saline). Pairwise comparisons indicated no significant differences from WT *vs OErsmA* (*P* = 0.1924).
(TIF)
