# Supplementary figures and images for: RsmA Regulates Aspergillus fumigatus Gliotoxin Cluster Metabolites Including Cyclo(L-Phe-L-Ser), a Potential New Diagnostic Marker for Invasive Aspergillosis
Source: PLoS One. 2013 May 6;8(5):e62591. doi: 10.1371/journal.pone.0062591 (PMC3646020; doi:10.1371/journal.pone.0062591)

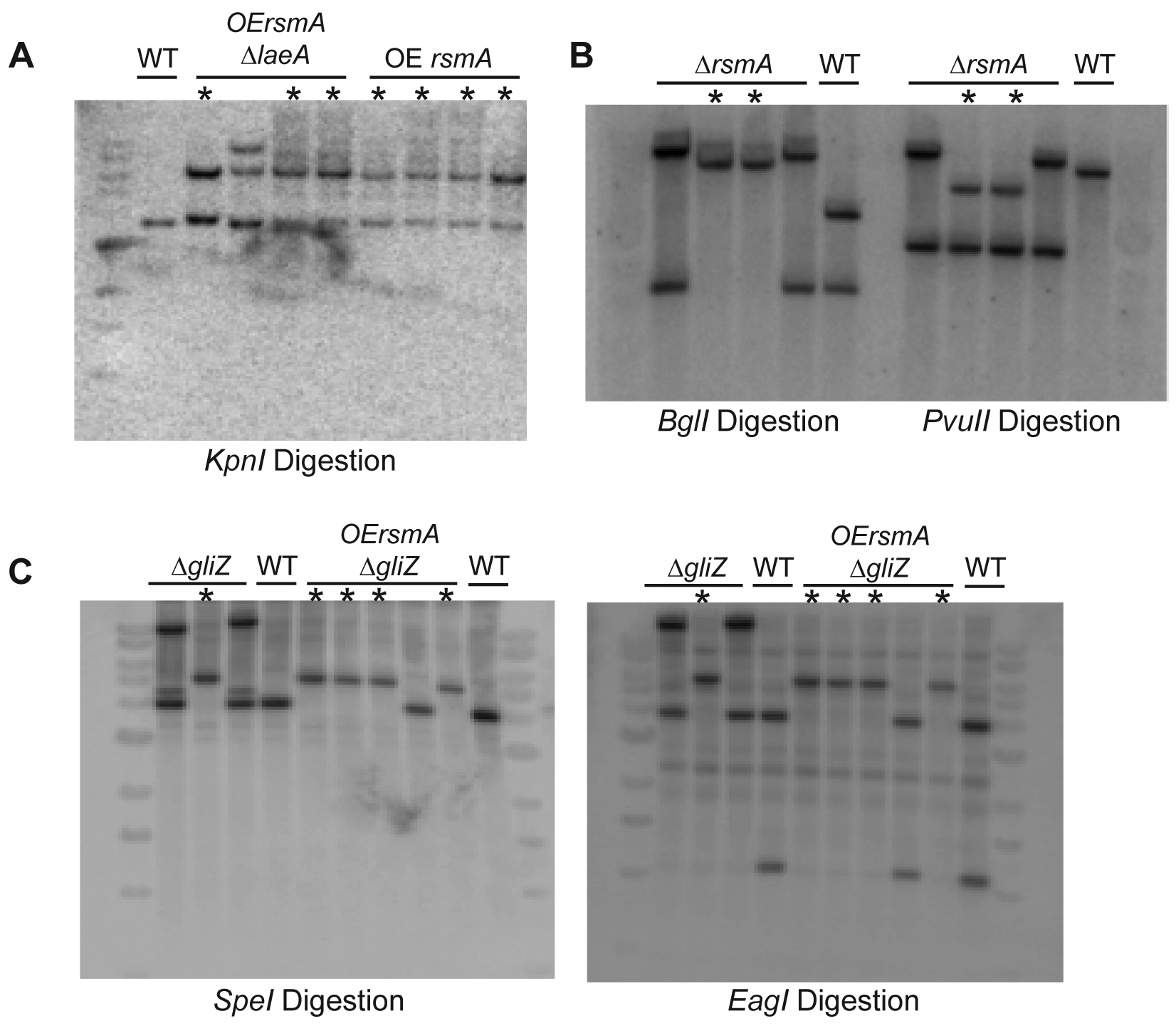

Supplement: Figure S1 — Overexpression and deletion of rsmA , and deletion of a transcription factor of gliotoxin biosynthesis, gliZ. A. Southern blot analysis of the OErsmAΔlaeA, OErsmA and wild type (WT) strains. Genomic DNA was digested with KpnI. Expected hybridization band patterns: 3.5 kb and 5.6 kb for mutants, and 3.5 kb for wild type strain. B. Southern blot analysis of the ΔrsmA mutant strains and wild type (WT) strain. Genomic DNA was digested with BglI and PvuII. Expected hybridization band patterns: 5.572 kb (BglI), 4.571 kb and 2.457 kb (PvuII) for mutants; 1.633 kb and 3.149 kb (BglI), 6.038 kb (PvuII) for wild type strain. C. Southern blot analysis of the ΔgliZ and OErsmAΔgliZ mutant strains and wild type(WT) strain. Genomic DNA was digested with SpeI and EagI. Expected hybridization band patterns: 5.17 kb (SpeI) and 6.21 kb (EagI) for mutants; 4.03 kb (SpeI), 1.13 kb and 3.96 kb (EagI) for wild type strain. The arrowheads denote the correct mutants. (TIF) [file pone.0062591.s001.tif]

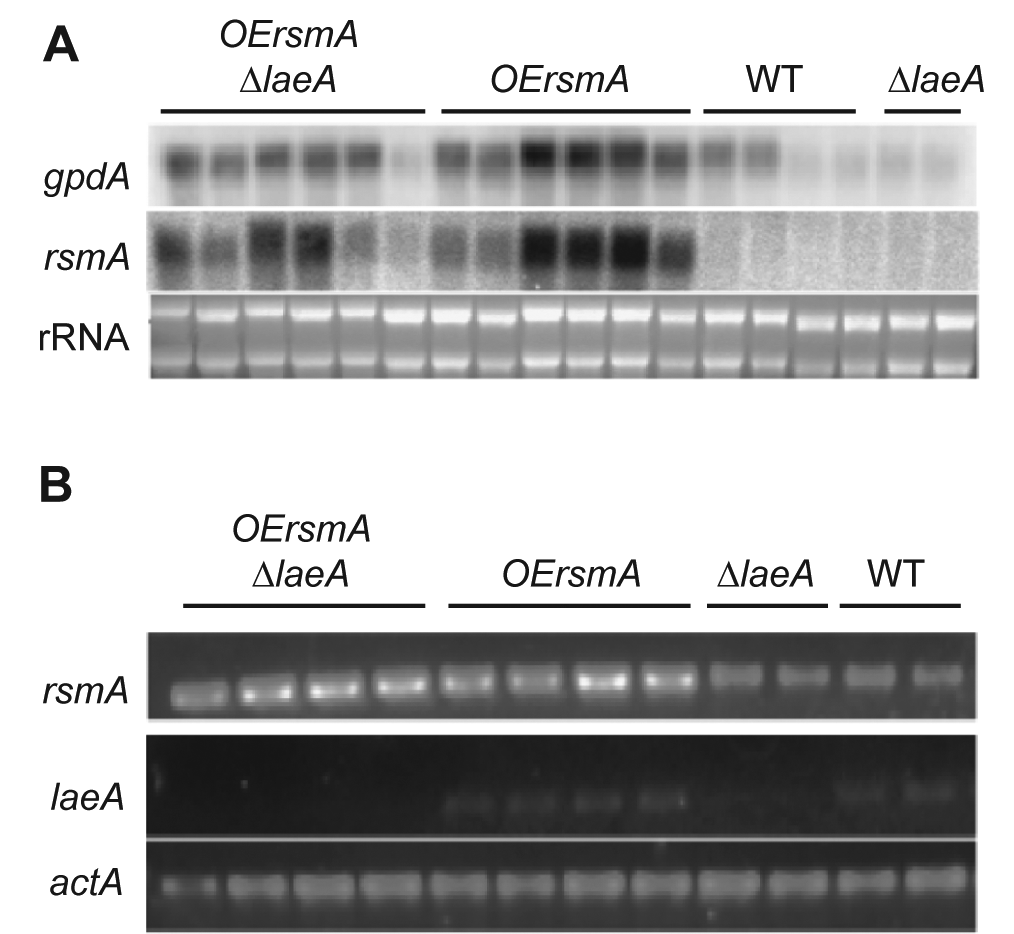

Supplement: Figure S2 — Detection of rsmA by Northern blot and reverse transcriptase- PCR (RT-PCR). A. Northern blot analysis of A. fumigatus wild type (WT), ΔlaeA, OErsmAΔlaeA and OErsmA strains. 107 spores/ml were inoculated in triplicates into liquid GMM and incubated at 37°C shaking for 48 h. Mycelia were collected, frozen in liquid nitrogen and lyophilized overnight. Total RNA was isolated. An internal fragment of the rsmA open reading frame was used as a probe. As a control, RNA blots were also hybridized with an internal fragment of the gpdA gene. B. Reverse transcriptase-PCR of wild type (WT), ΔlaeA, OErsmAΔlaeA and OErsmA strains. RNA of each strain was extracted from freeze-dried mycelia, treated with DNase1, reverse transcribed and the resultant cDNA was amplified with primers specific for rsmA, laeA and actin (serves as a control). (TIF) [file pone.0062591.s002.tif]

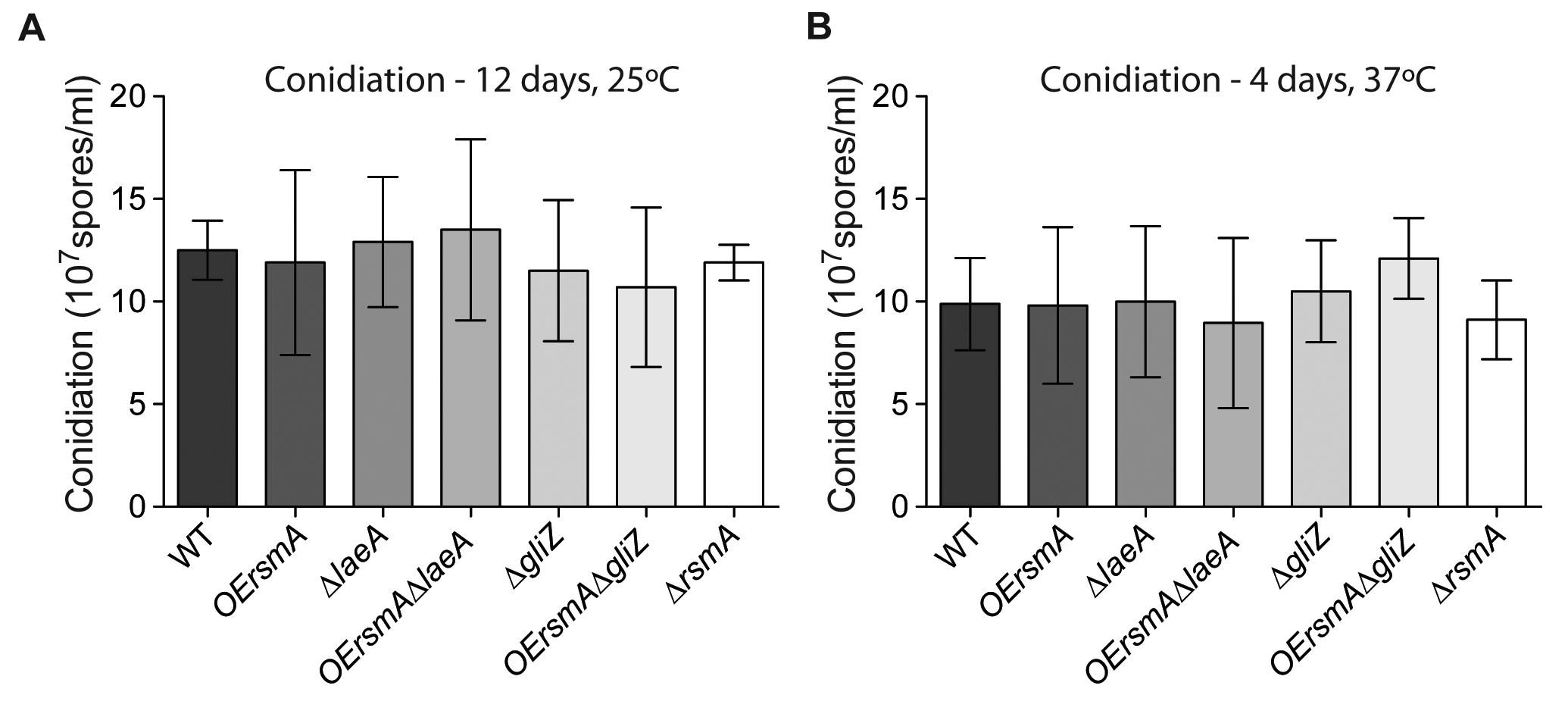

Supplement: Figure S3 — Average sporulation of A. fumigatus strains. 104 conidia of each strain were point inoculated on GMM and grown under dark conditions at 25°C for 12 days (A) and at 37°C for 4 days (B). Agar plugs were extracted from the center of the colonies, homogenized in water and spores counted using a haemocytometer. Means ± standard errors are indicated for four replicates of each strain. Levels not connected by same letter are significantly different (P>0.15) according to the student’s t test. (TIF) [file pone.0062591.s003.tif]

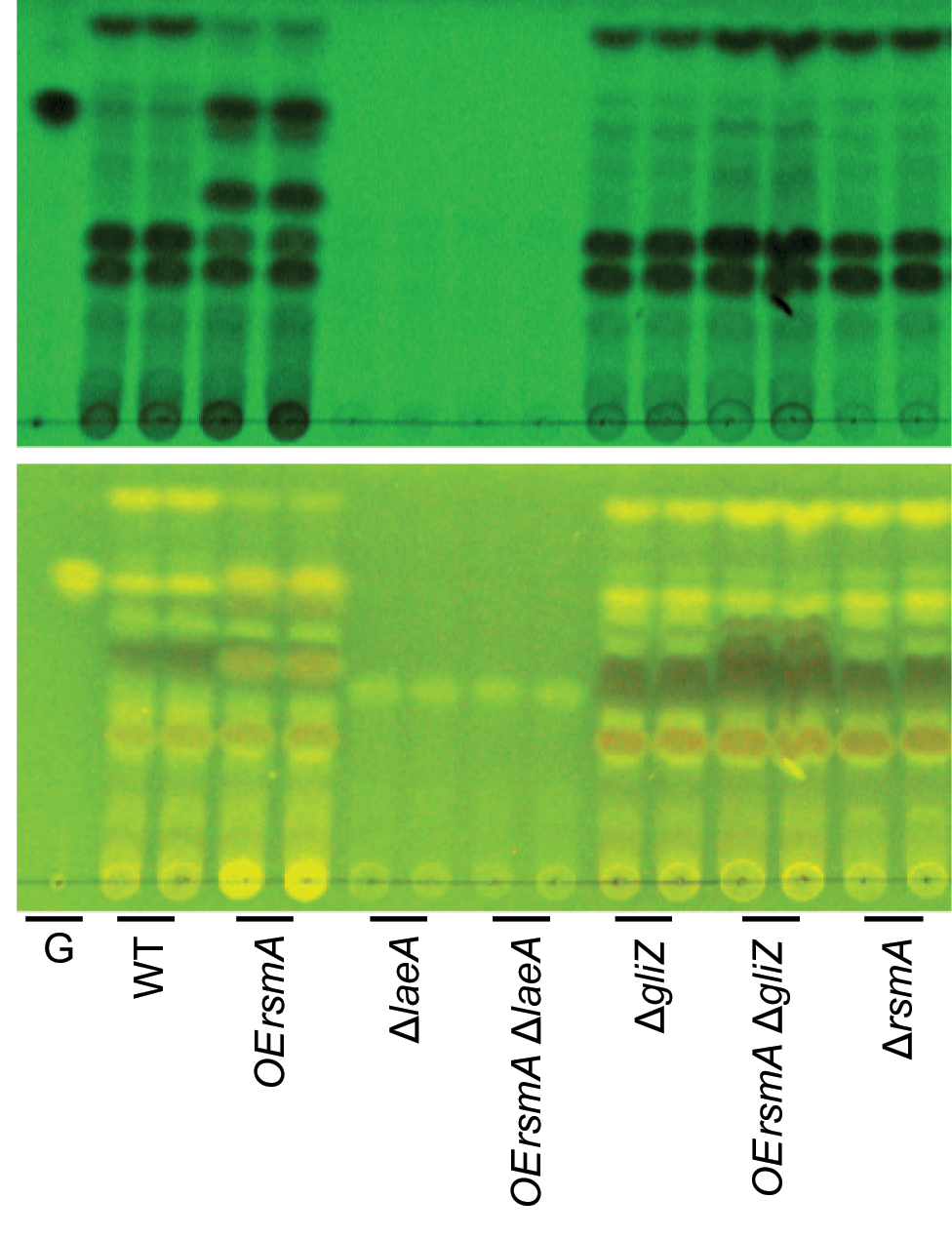

Supplement: Figure S4 — Thin-layer chromatography profiles of secondary metabolites produced by A. fumigatus wild type (WT), OE rsmA , ΔlaeA , OE rsmAΔlaeA, ΔgliZ, OE rsmAΔgliZ and ΔrsmA strains. Secondary metabolites were extracted by chloroform from cultures grown in liquid GMM at 25°C, 280 rpm for 3 days. Dried extracts were resuspended in 100 µl of methanol, and 10 µl was used for separation on TLC plate. All strains were triplicated. The solvent condition was chloroform:acetone (7∶3), and the plates were visualized at 254 nm (top) and 366 nm (bottom). G, gliotoxin standard. (TIF) [file pone.0062591.s004.tif]

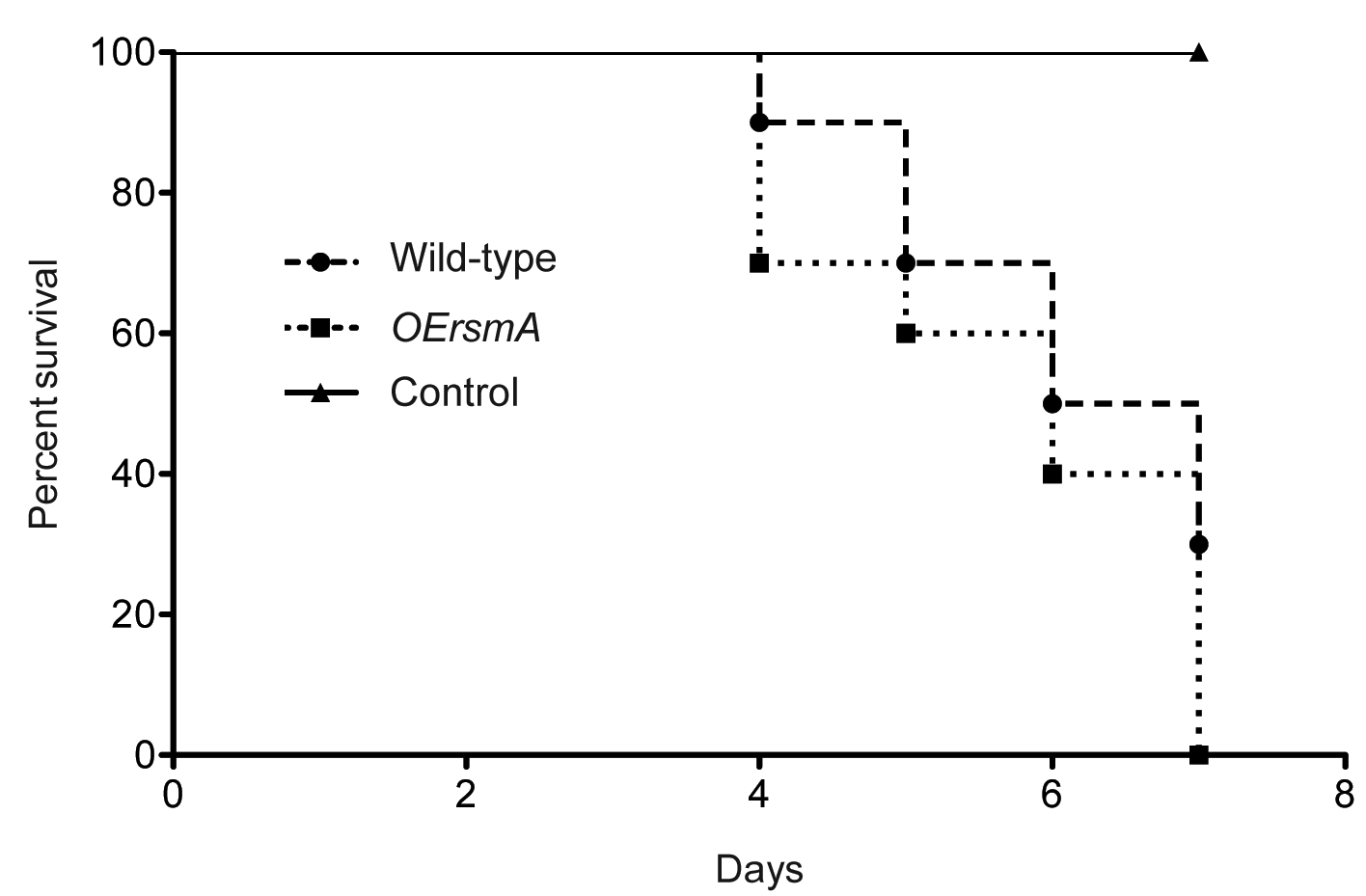

Supplement: Figure S5 — Virulence of OErsmA in a murine lung infection model. 10 female Swiss ICR mice immunosuppressed by intraperitoneal injection of cyclophosphamide (200 mg/kg) and cortisone acetate (250 mg/kg) were inoculated intranasally with 50 µl of 1×10^7^ conidia/ml of A. fumigatus wild type (AF293) and OErsmA and control (saline). Pairwise comparisons indicated no significant differences from WT vs OErsmA (P = 0.1924). (TIF) [file pone.0062591.s005.tif]
